# GABAergic neuron-specific whole-brain transduction by AAV-PHP.B incorporated with a new GAD65 promoter

**DOI:** 10.1186/s13041-021-00746-1

**Published:** 2021-02-15

**Authors:** Chiaki Hoshino, Ayumu Konno, Nobutake Hosoi, Ryosuke Kaneko, Ryo Mukai, Junichi Nakai, Hirokazu Hirai

**Affiliations:** 1 Department of Neurophysiology and Neural Repair, Gunma University Graduate School of Medicine, 3-39-22, Gunma, 371-8511 Japan; 2 Viral Vector Core, Gunma University Initiative for Advanced Research (GIAR), Gunma, 371-8511 Japan; 3 Bioresource Center, Gunma University Graduate School of Medicine, Gunma, 371-8511 Japan; 4 Osaka University, Graduate School of Frontier Biosciences, 1-3 Yamadaoka, Suita, Osaka 565-0871 Japan; 5 Department of Ophthalmology, Gunma University Graduate School of Medicine, 3-39-22, Gunma, 371-8511 Japan; 6 Division of Oral Physiology, Tohoku University Graduate School of Dentistry, Sendai, 980-8575 Japan

**Keywords:** Interneuron, Inhibitory neuron, Chandelier cell, GAD65, Dlx, GABA, Promoter, AAV, AAV-PHP.B

## Abstract

GABAergic interneurons play a critical role in tuning neural networks in the central nervous system, and their defects are associated with neuropsychiatric disorders. Currently, the mDlx enhancer is solely used for adeno-associated virus (AAV) vector-mediated transgene delivery into cortical interneurons. Here, we developed a new inhibitory neuron-specific promoter (designated as the mGAD65 promoter), with a length of 2.5 kb, from a mouse genome upstream of exon 1 of the *Gad2* gene encoding glutamic acid decarboxylase (GAD) 65. Intravenous infusion of blood–brain barrier-penetrating AAV-PHP.B expressing an enhanced green fluorescent protein under the control of the mGAD65 promoter transduced the whole brain in an inhibitory neuron-specific manner. The specificity and efficiency of the mGAD65 promoter for GABAergic interneurons, which was assessed at the motor cortex, were almost identical to or slightly higher than those of the mDlx enhancer. Immunohistochemical analysis revealed that the mGAD65 promoter preferentially transduced parvalbumin (PV)-expressing interneurons. Notably, the mGAD65 promoter transduced chandelier cells more efficiently than the mDlx enhancer and robustly labeled their synaptic boutons, called the cartridge, targeting the axon initial segments of excitatory pyramidal neurons. To test the ability of the mGAD65 promoter to express a functional molecule, we virally expressed G-CaMP, a fluorescent Ca^2+^ indicator, in the motor cortex, and this enabled us to monitor spontaneous and drug-induced Ca^2+^ activity in GABAergic inhibitory neurons. These results suggest that the mGAD65 promoter is useful for AAV-mediated targeting and manipulation of GABAergic neurons with the dominance of cortical PV-expressing neurons, including chandelier cells.

## Introduction

Inhibitory interneurons constitute 10–20% of all neurons in the cerebral cortex [1–3]. They have numerous neurochemical, morphological, and functional classes. Recent studies have shown that they belong to one of three groups defined by the expression of the Ca^2+^-binding protein parvalbumin (PV), neuropeptide somatostatin (SST), and the ionotropic serotonin receptor 5HT3a (5HT3aR) [1]. The PV-expressing cells, SST-expressing cells, and 5HT3aR-expressing cells accounted for ~ 40%, ~ 30%, and ~ 30% of the cortical GABAergic neuron population, respectively. The most abundant PV group includes fast-spiking basket cells and chandelier cells, whereas the SST group is dominated by Martinotti cells [4, 5]. The 5HT3aR group includes all of the neurons that express the neuropeptide VIP, which accounts for approximately 40% of this group as well as the remaining 60% of neurons that do not express VIP.

The PV-expressing basket cell, the most abundant subtype, which accounts for approximately 30% of non-pyramidal neurons, is responsible for the perisomatic inhibition of excitatory pyramidal neurons [6]. The second-largest cell is the Martinotti cell, and its axons ascend to layer 1 and form synapses with the dendritic tufts of pyramidal neurons [5, 6]. Chandelier cells, which account for a small proportion, demonstrate distinctive axonal arborization. The beads of axon terminals, also called cartridges, vertically innervate the axon initial segments (AISs) of pyramidal cells [7]. Recent studies have revealed that cortical interneuron activity plays a crucial role in shaping excitatory circuits during learning and memory expression [6, 8], and dysfunction of inhibitory neuron subtypes is thought to underlie various aspects of psychiatric disorders [6, 7, 9, 10].

Recent studies have developed several cell type-specific promoters that can be incorporated into viral vectors to enable cell-type-specific transgene expression. These promoters include neuron-specific synapsin I promoter [11], astrocyte-specific glial fibrillary acidic protein (GFAP) promoter [12], and cerebellar Purkinje cell-specific L7-6 promoter [13]. The Ca^2+^/calmodulin-dependent protein kinase II (CaMKII) promoter has been used as a forebrain-predominant excitatory neuron-specific promoter [14, 15], while two different studies identified an inhibitory neuron-specific distal-less homeobox (*Dlx*) gene enhancer sequence, which also confined transgene expression to the forebrain [16, 17]. In this study, we report a novel inhibitory neuron-specific promoter. Intravenous infusion of the blood–brain barrier (BBB)-penetrating AAV-PHP.B-expressing enhanced green fluorescent protein (GFP) under the control of the new promoter achieved highly efficient and widespread labeling of inhibitory neurons throughout the brain. We have described the distinct features of the new promoter in comparison to the currently available inhibitory neuron-specific Dlx enhancer.

## Materials and methods

### Animals

Wild-type and VGAT-tdTomato mice from C57BL/6 backgrounds [18] were used in this study. All procedures for the care and treatment of animals were performed according to the Japanese Act on the Welfare and Management of Animals and the Guidelines for Proper Conduct of Animal Experiments issued by the Science Council of Japan. The experimental protocol was approved by the Institutional Committee of Gunma University (No. 18–019; 19–028). All efforts were made to minimize suffering and reduce the number of animals used.

### Viral vectors

**Fig. 1:**
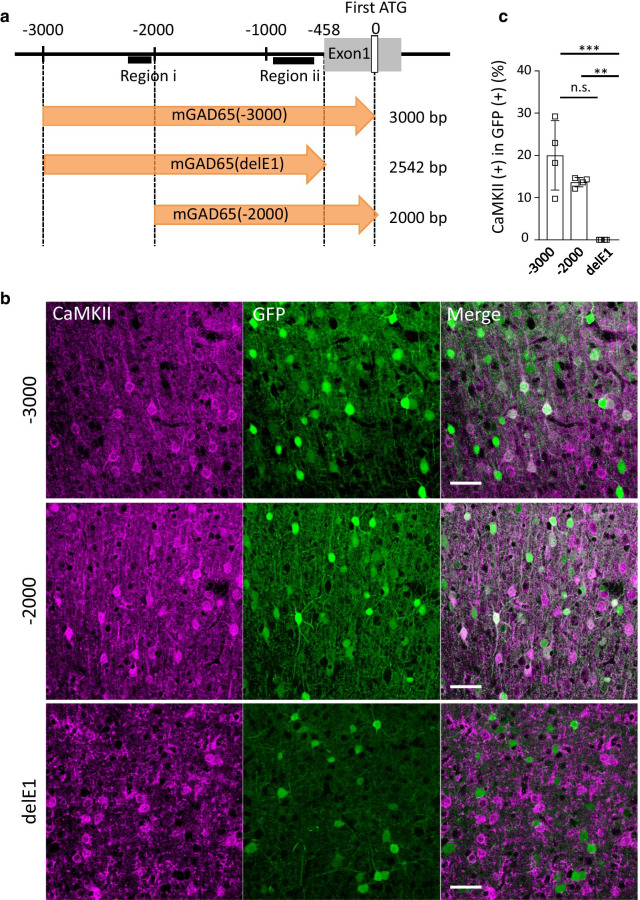
Inhibitory neuron-specific mGAD65 promoter candidates and their screening using immunohistochemistry. **a** Schematic displaying mouse *Gad2* (mGAD65) promoter regions examined as candidates for the inhibitory neuron-specific promoter. Mouse genomic regions (3000, 2542, and 2000 bp) upstream of the first ATG codon (position 0) in exon 1 of the *Gad2* gene were cloned from C57BL/6-mice cDNA library. mGAD65 (delE1) shares most portions with mGAD65 (-3000), but it lacks the exon 1 sequence. Black bars (Regions i and ii) depict relative positions of the TAAT/ATTA motif, a core sequence for homeodomain protein binding. **b** Immunohistochemistry of the motor cortices obtained from mice that received intravenous infusions of AAV-PHP.eB expressing GFP by one of the promoters described in **a**. The brain sections were double immunolabeled for GFP and CaMKII, a marker of the excitatory neurons in the cerebral cortex. Scale bars; 50 μm. **c** Quantitative analysis of each promoter candidate in terms of the specificity for inhibitory neurons. Ratios of CaMKII-expressing (double-positive) excitatory neurons to total GFP ( +) cells were measured. For respective promoter candidates, we examined 4 visual fields in 2 sections from one animal. The value obtained from each visual field was plotted. We found 29 double-positive cells in 147 GFP ( +) cells (20.0 ± 3.6%) for the mGAD65 (− 3000), and 18 double-positive cells in 131 GFP ( +) cells (13.7 ± 0.5%) for the mGAD65 (− 2000); no double-positive cells were found in 75 GFP ( +) cells (0.0 ± 0.0%) for the mGAD65 (delE1). ***p = 0.0007, **p = 0.0089 and *p* = 0.28 by ANOVA with Bonferroni’s post hoc test

We used BBB-penetrating AAV-PHP.B [19], except for Fig. 1, which used AAV-PHP.eB [20]. The expression plasmid pAAV, comprising a polyadenylation signal sequence and woodchuck hepatitis virus post-transcriptional regulatory element (WPRE), was designed to express GFP under the control of a mouse genomic region upstream of the mouse *Gad2* gene encoding glutamic acid decarboxylase (GAD) 65 (see Fig. 1a for detail) or the mouse *Dlx* gene enhancer (previously referred to as I56i [21] or mI56i [22]) combined with a minimal promoter (mDlx enhancer, hereafter) [16]. The mGAD65 promoter and mDlx enhancer were inserted into the pAAV plasmid upstream of the GFP gene at restriction enzyme sites for XhoI and AgeI. pAAV-PHP.B and pAAV-PHP.eB were constructed from the pAAV2/9 plasmid [19], which was provided by Dr. James M. Wilson at the University of Pennsylvania. Recombinant single-stranded AAV-PHP.B/PHP.eB vectors were produced by co-transfection into HEK293T cells (HCL4517; Thermo Fisher Scientific; Waltham, MA, USA) with three plasmids: the expression plasmid, pHelper (Stratagene, La Jolla, CA, USA), and a packaging plasmid (pAAV-PHP.B or pAAV-PHP.eB) as previously described [13, 23]. Briefly, HEK293T cells, which were cultured in Dulbecco’s modified Eagle’s medium (D-MEM; D5796-500Ml, Sigma-Aldrich, St Louis, MO, USA) supplemented with 8% fetal bovine serum (Sigma-Aldrich), were transfected with three plasmids: expression plasmids [pAAV/mGAD65-EGFP-WPRE-SV40, pAAV/mDlx enhancer-EGFP-WPRE-SV40, pAAV/mDlx enhancer-tdTomato-WPRE-SV40, or pAAV/mGAD65-GCaMP-WPRE-SV40], pHelper (Stratagene, La Jolla, CA, USA), and pAAV-PHP.B/PHP.eB using polyethylenimine. Viral particles were harvested from the culture medium 6 days after transfection and concentrated by precipitation with 8% polyethylene glycol 8000 (Sigma-Aldrich) and 500 mM sodium chloride. The precipitated AAV–PHP.B/PHP.eB particles were resuspended in D-PBS and purified with iodixanol (OptiPrep; Axis-Shield Diagnostics, Dundee, Scotland) continuous gradient centrifugation. The viral solution was further concentrated in D-PBS using Vivaspin 20 (100,000 MWCO PES, Sartorius, Gottingen, Germany). The genomic titers of the viral vector were determined by real-time quantitative PCR using THUNDERBIRD SYBR qPCR Mix (Toyobo, Osaka, Japan) using the 5′-CTGTTGGGCACTGACAATTC-3′ and 5′-GAAGGGACGTAGCAGAAGGA-3′ primers, which targeted the WPRE sequence. The expression plasmid was used as the standard.

### Intravenous injection

Eight-to fourteen-week-old mice were used in this study. After inducing deep anesthesia via intraperitoneal injection of ketamine (100 mg/kg BW) and xylazine (10 mg/kg BW), 100 μL of AAV-PHP.B (5.0 × 10^13^ vg/mL) or AAV-PHP.eB (3.0 × 10^13^ vg/mL) was injected into the orbital sinus using a 0.5 ml syringe with a 30-gauge needle (08,277; Nipro, Osaka, Japan) for 30 to 40 s. For Additional file 2: Figure S2, equal titers (5.0 × 10^12^ vg) of AAV-PHP.B-expressing tdTomato under the control of the mDlx enhancer and AAV-PHP.B expressing GFP under the control of the mGAD65 promoter were mixed in advance and injected as described above.

### Direct cerebellar injection

The AAV-PHP.B was injected directly into the cerebellar tissue. After inducing deep anesthesia, the mice were placed in a stereotactic frame. The skin covering the occipital bone was cut, and a burr hole was made 7 mm caudal to the bregma. The tip of a Hamilton syringe (33 gauge) with an attached micropump (UltraMicroPump II; World Precision Instrument (WPI) Sarasota, FL, USA) was inserted 1.8 mm below the pia mater of the cerebellar vermis. Ten microliters of viral solution (1 × 10^13^ vg/mL) was injected at a rate of 400 nL/min using a microprocessor-based controller (Micro4; WPI).

### Immunohistochemistry

Depending on the antibodies used, we employed different immunohistochemistry protocols (see Additional file 1: Table S1 for details). Three weeks after the injection of AAV-PHP.B vectors, the mice were deeply anesthetized and perfused intracardially with 4% paraformaldehyde phosphate buffer (pH 7.4). Their brains were removed and immersed in 4% paraformaldehyde in 0.1 M phosphate buffer at 4 °C. Floating (50 μm thick) and cryostat (20 μm thick) brain sections were prepared using a vibratome (VT1000S, Leica, Wetzlar, Germany) and cryostat (CM3050S, Leica), respectively. The slices were permeabilized, blocked with an appropriate blocking solution, and treated with blocking solution containing the following antibodies: mouse monoclonal anti-CaMKII (1:100; 05–532; Merck, Germany), rat monoclonal anti-GFP (1:1000; 04,404–84; Nacalai, Kyoto, Japan), rabbit polyclonal anti-GABA (1:1000; A2052; Sigma-Aldrich), goat polyclonal anti-PV (1:200; PV-Go-Af460; Frontier Institute, Hokkaido, Japan), rat monoclonal anti-SST (1:100; MAB354; Merck, Germany), goat polyclonal anti-ankyrin G (P-20) (1:50, SC-31778, Santa Cruz Biotechnology, Dallas, TX, USA), mouse monoclonal anti-calbindin D-28 k (1:500; Swant, Bellinzona, Switzerland), or mouse monoclonal anti-mGluR2 (1:1000; ab15672; Abcam, Cambridge, UK). After rinsing several times with PBS or PBS containing Triton X-100 at room temperature (24–26 °C), the slices were incubated with the relevant secondary antibodies (Thermo Fisher Scientific) (see Additional file 1: Table S1). They were mounted on glass slides after rinsing several times with PBS or PBS containing Triton X-100 at room temperature (24–26 °C). For mounting medium, we used Prolong Gold/Diamond Antifade Reagent (Thermo Fisher Scientific) and CC/Mountant antifade reagent (Diagnostic BioSystems, Pleasanton, CA, USA) for floating and cryostat sections, respectively.

### Retinal slice

Mice treated with AAV-PHP.B were anesthetized and fixed as described above. Their eyes were removed and frozen in Tissue-Tek O.C.T. compound (Sakura Finetek Japan, Tokyo, Japan). The 16-μm-thick retinal slices were prepared using a cryostat (CM3050S, Leica) and mounted on glass slides with Prolong Diamond Antifade Reagent (Thermo Fisher Scientific).

### Confocal microscopy

Most of the fluorescent images of the brain and retinal sections were acquired using a laser-scanning confocal microscope (LSM 800, Carl Zeiss, Oberkochen, Germany) with 20 × or 40 × objectives, and z-stack images of different focal planes were generated. The acquired images were combined using ImageJ software with the MosaicJ plugin [24].

### Screening of the promoter candidates

Sagittal brain slices (50 μm thickness) were obtained from mice treated with AAV-PHP.eB expressing GFP by the mGAD65 promoter candidate (3 k, 2 k, or delE1). The slices were immunolabeled for CaMKII, a marker of excitatory neurons. Fluorescent images were obtained using a confocal microscope with a 20 × magnification. We determined the ratio of cells double-positive for GFP and CaMKII (excitatory neurons) to the total number of GFP-expressing cells.

### Relative GFP intensity in the whole brain

Bright-field and native GFP fluorescence images of the whole brain were acquired using a fluorescence stereoscopic microscope (VB-700; Keyence, Osaka, Japan). To measure the GFP fluorescence intensity of the forebrain and hindbrain, the margin of the corresponding part of the brain was traced on the bright-field image, except for the olfactory bulb, superior and inferior colliculi, and brainstem. GFP fluorescence intensity within the enclosed area was measured using the ImageJ software (Fiji). Autofluorescence of the brain was measured in six non-injected VGAT-tdTomato mice, and the average value was subtracted from the GFP fluorescence values of all the samples. Finally, data were normalized to the mean value (100) from the forebrains of VGAT-tdTomato mice that received AAV-PHP.B expressing GFP under the control of mDlx enhancer.

### Specificities and efficiencies of the mGAD65 promoter and mDlx enhancer

To determine the specificity and efficiency of the mGAD65 promoter and mDlx enhancer, GFP and tdTomato fluorescent images were obtained from sagittal brain slices (50 μm thickness) using a fluorescent microscope (BZ-X700; Keyence) with a 20 × objective. The number of GFP ( +) and/or tdTomato ( +) cells was counted manually.

### Proportion of interneuron subtypes in cortical GABAergic interneurons

To measure the ratio of PV ( +) or SST ( +) cells to transduced GABAergic interneurons in the cerebral cortex, wild-type mice were intravenously infused with AAV-PHP.B expressing GFP under control of the mGAD65 promoter. Three weeks after the viral injection, sagittal brain slices (50 μm thickness) were immunolabeled for PV and SST. Fluorescent images were obtained using a confocal microscope with a 40 × magnification. We assessed the ratio of PV ( +) or SST ( +) cells to GFP ( +) cells and the proportion of GFP ( +) cells in PV ( +) or SST ( +) cells.

### Confocal Ca^2+^ imaging in cortical GABAergic interneurons with G-CaMP

For the direct injections of AAV-PHP.B expressing G-CaMP7.09 under the control of the mGAD65 promoter, the mice were kept under stable anesthesia with isoflurane (0.5%) until the end of surgery. The anesthetized mice were fixed on a stereotactic frame (SRS-5-HT; Narishige, Tokyo, Japan) with a stereotaxic micromanipulator (SMM-100; Narishige). Their scalps were cut and holes through the occipital bone were made at the stereotaxic coordinate of AP + 1.0 mm and ML ± 1.0 mm. The tip of a Hamilton syringe (10 μL, 33 gauge; 701SN 33/2"/PT3), with an attached microinjector (IMS-20, Narishige), was stereotactically inserted into the cerebral cortex at a depth of 0.6–0.8 mm from the cranial bone surface. Five microliters of the viral solution (2 × 10^13^ vg/mL) was injected at a rate of 83.3 nl/min.

Confocal Ca^2+^ imaging of the acute cortical brain slices was performed as described previously [25] with some modifications. G-CaMP7.09 (G-CaMP) is a recently developed gene-encoded Ca^2+^ indicator that increases its fluorescence (similar wavelength range to GFP) when intracellular Ca^2+^ concentration is elevated [26]. AAV-injected adult VGAT-tdTomato mice were utilized 3 weeks after the injections to ensure a stable G-CaMP expression. Coronal brain slices (200–300 µm in thickness) of the mouse motor cortex were prepared using a vibroslicer (VT1200S; Leica, Germany) and maintained in an ACSF containing 125 mM NaCl, 2.5 mM KCl, 2 mM CaCl_2_, 1 mM MgCl_2_, 1.25 mM NaH_2_PO_4_, 26 mM NaHCO_3_, and 10 mM D-glucose and bubbled with 95% O_2_ and 5% CO_2_ at room temperature (24–26 °C) for more than 1 h before the imaging. To examine Ca^2+^ signals from GABAergic interneurons, we selected G-CaMP ( +) and tdTomato ( +) double-positive cells for imaging. The imaging areas were restricted to the M1-M2 regions (mostly layers 2 to 3) of the motor cortex. Confocal fluorescence images of G-CaMP signals were acquired every 0.1 s (100 ms exposure time, 512 × 512 pixels, no binning) with a 40 × water immersion objective (LUMPLFLN 40XW, Olympus, Tokyo, Japan), a water-cooled CCD camera (iXon3 DU-897E-CS0-#BV-500, Andor, Belfast, Northern Ireland), and a high-speed spinning-disk confocal unit (CSU-X1, Yokogawa Electric, Tokyo, Japan) attached to an upright microscope (BX51WI, Olympus, Tokyo, Japan). A 488-nm light beam from a diode laser module (Stradus 488–50, VORTRAN, USA) for G-CaMP imaging and a 561-nm laser (LDSYS-561GH-SP43, Solution Systems, Japan) for tdTomato imaging were used for excitation, and the emitted fluorescence was collected through a band-pass filter (500–550 nm for G-CaMP and 580–660 nm for tdTomato). During the recordings, cortical slices were perfused with ACSF bath solution at room temperature (24–26 °C).

To stimulate GABAergic interneurons, 10 µM glutamate (Glu) dissolved in ACSF bath solution or high K^+^ solution (total 12.5 mM KCl in the ACSF bath solution) was applied extracellularly via a gravity-fed bath-application device. Image processing and analysis were performed with Andor iQ2 (Andor), NIH imageJ, Igor Pro8 (WaveMetrics), and custom-written programs by NH. Image drift (translation drift) was sometimes observed. In such cases, the drifted images were corrected using ImageJ plugins (template matching and slice alignment using the OpenCV library) provided by Q. Tseng (https://sites.google.com/site/qingzongtseng/template-matching-ij-plugin#downloads). Fluorescence at time t (Ft) in each pixel was background-subtracted, and the Ca^2+^-dependent relative change in fluorescence was calculated using the following formula: ΔF/F_base_, where F_base_ is the basal fluorescence intensity averaged during pre-stimulus frames (i.e., ~ 100 frames before stimulation) and ΔF = Ft—F_base_. The background fluorescence was obtained from a region lacking a cell structure in the same frame. The mean values of ΔF/F_base_ in each region of interest (ROI) were calculated for each frame. ROIs were placed on the tdTomato-positive and G-CaMP-positive cellular structures (usually soma-like structures). To quantify Glu- or high K-induced Ca^2+^ signals in the G-CaMP positive cells, the peak amplitude of ΔF/F_base_ was measured within a time window of ~ 100 s after the bath application onset because the drug took tens of seconds to reach the recorded cells (Fig. 7e and f).

### Statistical analysis

GraphPad PRISM version 7 (GraphPad Software, San Diego, CA, USA) was used for the statistical analysis and production of graphic images, except the Ca^2+^ imaging data.

Statistical methods are shown in the text and/or in each figure legend. The data are expressed as mean ± standard error of the mean.

## Results

### Identification of a candidate region for inhibitory neuron-specific promoters

Inhibitory neurons release the key inhibitory neurotransmitter, GABA, which is synthesized from glutamate by glutamic acid decarboxylase (GAD); GAD is composed of two isoforms, namely GAD67 and GAD65, which are encoded by the *Gad1* and *Gad2* genes in the mouse, respectively [27]. Therefore, we examined *Gad* promoter regions for inhibitory neuron-specific promoters. A previous study revealed pivotal roles of *Dlx1* and *Dlx2*, members of the *Dlx* gene, in GABA synthesis [28]. Recently, Le et al. demonstrated that DLX1 and DLX2 directly bind to and activate the transcription of *Gad* promoters [29]. Moreover, using chromatin immunoprecipitation (ChIP) of the embryonic forebrain, they identified two Dlx target sequences within the *Gad2* promoter region. The Dlx target regions were at nucleotide positions of − 2294 to − 2088 and − 958 to − 598 (region i and region ii, respectively) (Fig. 1a), both of which contained specific homeodomain DNA-binding tetranucleotide TAAT/ATTA motifs [29]. Thus, these regions were postulated to be critical for the *Gad2* promoter in terms of inhibitory neuron specificity and promoter activity.

The packaging limitation of the AAV vector is approximately 4.7 kb, including the inverted terminal repeat (ITR) sequences at both ends. Given the size of a reporter gene and/or gene of interest, candidate promoters should be less than 3 kb in length. Taking these into consideration, we cloned a 3 kb region upstream of the first ATG codon of *Gad2* [mGAD65(− 3000)] and a similar region that lacked exon 1 [mGAD65(delE1)] (2,542 bp) from the C57BL/6-mouse genome (Fig. 1a). Of note, these two candidate regions for inhibitory neuron-specific promoters involved both Dlx-binding sites. In addition, we prepared an additional candidate consisting of a 2 kb sequence upstream of the first ATG codon of *Gad2* [mGAD65(− 2000)], which lacked one Dlx target sequence (Region i) [mGAD65(− 2000)] (Fig. 1a). These three promoter regions were subcloned into the pAAV expression plasmid. BBB-penetrating AAV-PHP.eB expressing GFP under the control of one of the three promoter candidates was produced and intravenously injected into mature mice.

Three weeks after the viral injection, cerebral sections were produced, immunostained for CaMKII, an excitatory neuron marker, and examined by confocal microscopy. All three promoter candidates expressed robust GFP in cortical cells, but we found numerous CaMKII-positive excitatory neurons expressing GFP in cerebral slices from mice treated with AAV-PHP.eB carrying mGAD65(− 3000) and mGAD65(− 2000) (Fig. 1b). Quantitative analysis revealed 15–20% of GFP-labeled cells as CaMKII-positive excitatory neurons (Fig. 1c). In contrast, no GFP-labeled cells expressed CaMKII in the slices that expressed GFP under the control of mGAD65(delE1) (Fig. 1b, c). To examine whether the GFP ( +) cells expressed GABA, the sections were immunolabeled for GABA. As shown in Additional file 1: Figure S1, cells expressing GFP by mGAD65(delE1) were co-immunolabeled for GABA. We assessed 89 GFP ( +) cells from 2 sections and found 87 cells (97.8%) that were co-immunolabeled for GABA. With these promising findings, we focused on mGAD65(delE1) (called mGAD65 promoter hereafter) for further characterization.

### Efficient transduction throughout the brain after intravenous infusion of AAV-PHP.B carrying the mGAD65 promoter

**Fig. 2:**
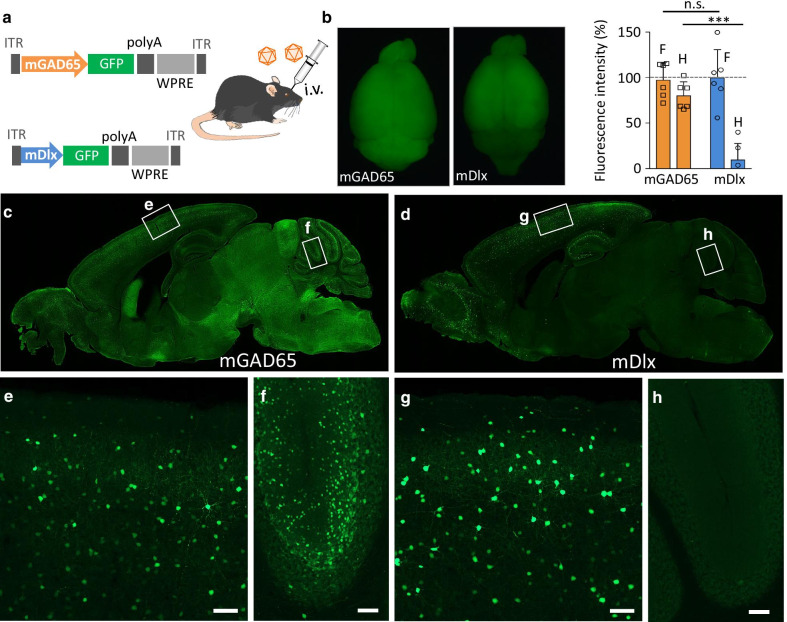
Native GFP of sagittal sections from mice 3 weeks after intravenous injection of AAV vectors. **a** Schematic showing the expression cassettes of AAV vectors, which express GFP under the control of the mGAD65 promoter or mDlx enhancer. The mice received intravenous injections (i.v.) of the blood–brain barrier (BBB)-penetrating AAV-PHP.B. **b** Fluorescent stereoscopy of whole brains expressing GFP by the mGAD65 (left) or mDlx enhancer (right). Graph next to the brains shows the relative GFP fluorescent intensities (average ± SEM) in the forebrain (**F**) and hindbrain (**H**) transduced by the mGAD65 promoter or mDlx enhancer. Values from respective mice (n = 6 mice in each group) were plotted on each bar graph. ****p < 0.0001 by 2-way ANOVA for repeated measures with Bonferroni’s posthoc test. **c**, **d** Sagittal brain sections from mice expressing GFP under the control of the mGAD65 promoter (**c**) or mDlx enhancer (**d**). **e**–**h** Enlarged GFP fluorescent images of square regions in panels (**c** and **d**). Scale bars; 100 μm

To examine whether the mGAD65 promoter works exclusively in inhibitory neurons, we injected the vesicular GABA transporter (VGAT)-BAC-based tdTomato-expressing mice (VGAT-tdTomato mice) with AAV-PHP.B-expressing GFP under the control of the mGAD65 promoter or mDlx enhancer (100 μL, 5.0 × 10^13^ viral genome/mL) (Fig. 2a). Because the VGAT-tdTomato mice expressed tdTomato specifically in inhibitory interneurons throughout the central nervous system (CNS) [18], we determined whether the transduced (GFP-expressing) cells were inhibitory GABAergic neurons without immunohistochemistry. The mice were sacrificed 3 weeks after viral injection. Fluorescent stereomicroscopy of the whole brain (Fig. 2b, left two images) showed robust transduction throughout the brain by the mGAD65 promoter, in contrast to the forebrain-restricted transduction by the mDlx enhancer. We measured GFP fluorescence intensity in the cerebral region (forebrain) and cerebellar cortex (hindbrain). The statistical analysis (two-way ANOVA for repeated measures, followed by Bonferroni's post hoc test) of GFP fluorescence intensities of the mGAD65 promoter and mDlx enhancer revealed no difference in the cerebral cortex (p > 0.9999), whereas there was a significant difference in the cerebellar cortex (p < 0.0001) (Fig. 2b, right graph). Subsequently, we obtained sagittal sections of virally treated brains (Fig. 2c, d). Enlarged images confirmed efficient GFP expression in both the cerebral and cerebellar cortices under the control of the mGAD65 promoter (Fig. 2e, f). Similar GFP expression was observed in the cerebral cortex, but not in the cerebellar cortex, in mice expressing GFP under the control of the mDlx enhancer (Fig. 2g, h).

### Inhibitory neuron-specific transduction by the mGAD65 promoter in the cerebral cortex

**Fig. 3:**
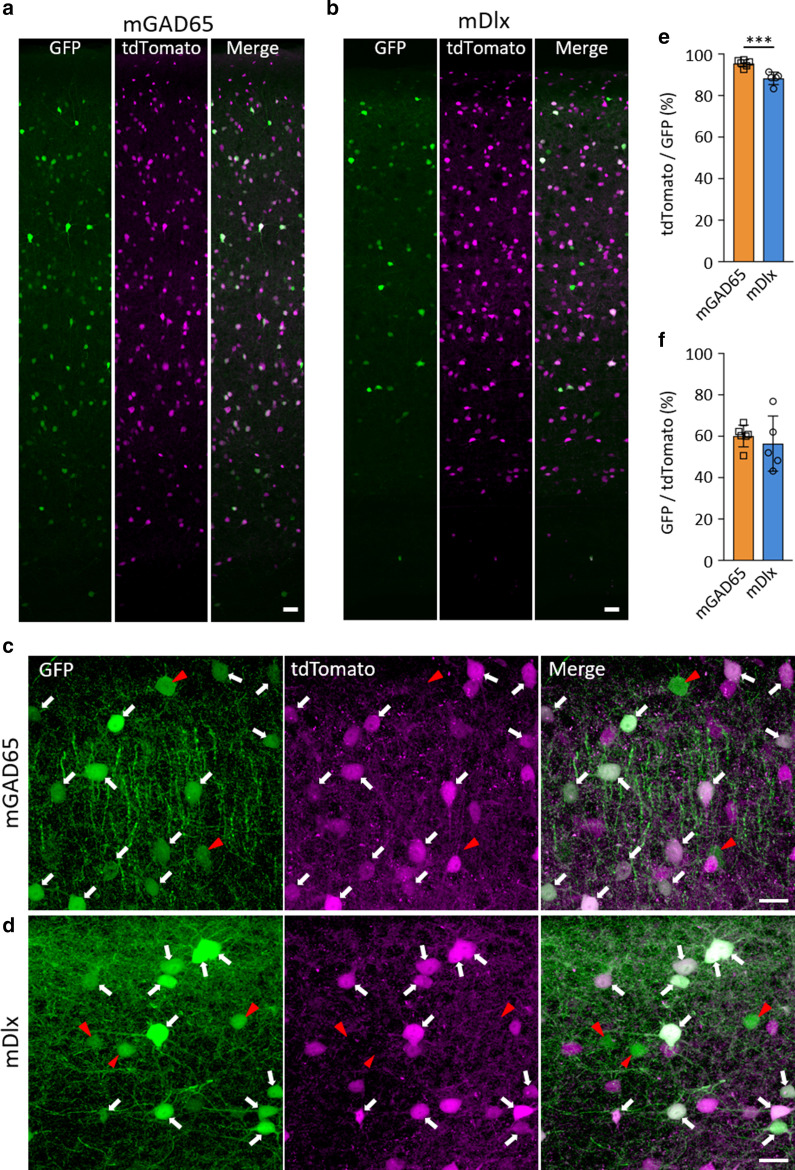
Specific and efficient transduction of cortical GABAergic interneurons by the mGAD65 promoter. VGAT-tdTomato mice that express tdTomato specifically in GABAergic neurons received intravenous (i.v.) injections of blood–brain barrier (BBB)-penetrating AAV-PHP.B expressing GFP under the control of the mGAD65 promoter or mDlx enhancer. The motor cortex was observed 3 weeks after the viral injection by confocal microscopy. **a**-**d** Low (**a**, **b**) and high (**c**, **d**) magnified images of native GFP and tdTomato. Arrows indicate cells double-labeled with GFP and tdTomato (GABAergic interneurons). Red arrowheads show GFP expression in tdTomato-negative cells (non-GABAergic cells). Scale bars; 50 μm (**a**, **b**) and 20 μm (**c**, **d**). **e** Graph showing highly specific transduction of cortical interneurons by the mGAD65 promoter. The specificity was determined by the ratio of double-labeled cells to total GFP ( +) cells. The value obtained from each mouse was plotted on the bar. For the mGAD65 promoter, we assessed 1553 GFP ( +) cells in 6 sections from 6 animals, and we found that 1483 cells were co-labeled with tdTomato (95.5 ± 0.7%). For the mDlx enhancer, we examined 1161 GFP ( +) cells in 5 sections from 5 animals, and we found that 1025 cells were co-labeled with tdTomato (88.3 ± 1.3%). ***p = 0.0007 by unpaired *t*-test. **f** Graph showing transduction efficiency of cortical interneurons by the mGAD65 promoter, in comparison to the mDlx enhancer. Transduction efficiency was determined by the proportion of GFP ( +) cells in tdTomato ( +) cells. For the mGAD65 promoter, we counted 2132 tdTomato ( +) cells in 6 sections from 6 animals, in which 1268 cells were GFP positive (60.1 ± 2.1%). For the mDlx enhancer, we observed 1503 tdTomato ( +) cells in 5 sections from 5 animals, in which 850 cells were GFP positive (56.5 ± 6.0%). *p* = 0.55 by unpaired *t*-test

We subsequently examined the specificity and efficacy of the mGAD65 promoter in inhibitory neurons. Low-power fields of the motor cortex showed an overlap of GFP with tdTomato in VGAT-tdTomato mice treated with AAV-PHP.B-expressing GFP under the control of mGAD65 (Fig. 3a), similar to those expressing GFP under the control of the mDlx enhancer (Fig. 3b). High-magnification images by confocal microscopy confirmed that the mGAD65 promoter and mDlx enhancer similarly expressed GFP in tdTomato-positive GABAergic interneurons in VGAT-tdTomato mice (arrows in Fig. 3c, d). Quantitative analysis showed that more than 95% of cells expressing GFP under the control of the mGAD65 promoter were co-labeled with tdTomato (95.5 ± 0.7%, n = 6 mice), whereas a significantly lower proportion of neurons expressing GFP under the control of the mDlx enhancer was tdTomato positive (88.3 ± 1.3%, n = 5 mice, *p* = 0.0007 by unpaired *t*-test) (Fig. 3e). The transduction efficiency of GABAergic neurons was assessed using the ratios of GFP and tdTomato double-labeled cells to tdTomato-positive interneurons. There was no significant difference in the transduction efficiencies of the mGAD65 promoter (60.1 ± 2.1%, n = 6 mice) and the mDlx enhancer (56.5% ± 6.0%, n = 5 mice) (*p* = 0. 55 by unpaired *t*-test) (Fig. 3f).

### PV (+) neuron-preferential transduction by AAV-PHP.B carrying the mGAD65 promoter

**Fig. 4:**
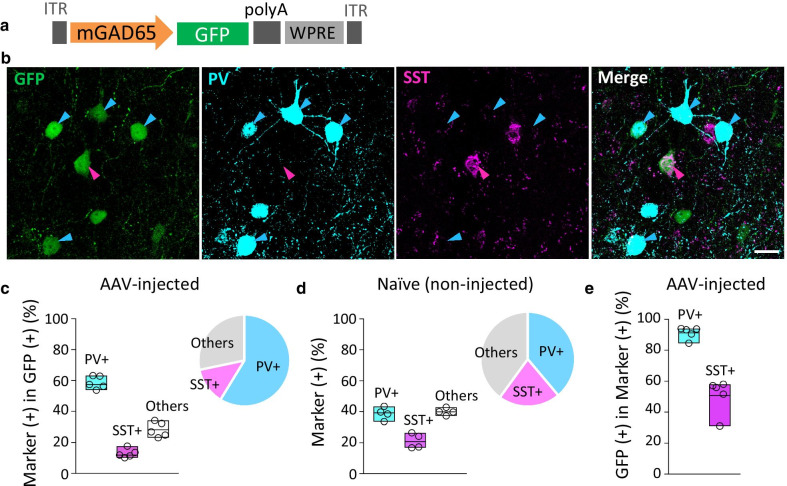
PV ( +) neuron-preferential transduction by the mGAD65 promoter. **a** The expression cassette of AAV-PHP.B expressing GFP under the control of the mGAD65 promoter. **b** Immunohistochemistry of the motor cortices from wild-type mice 3 weeks after the viral injection. Brain sections were immunostained for PV (light blue) and somatostatin (SST) (magenta). Blue arrowheads indicate GFP-expressing PV ( +) neurons, and magenta arrowheads show GFP-expressing SST ( +) neurons. Scale bar; 20 μm. **c** Bar and circle graphs showing proportions of PV ( +), SST ( +), and other cell types among GFP-expressing cells. We observed 2,884 GFP ( +) cells (n = 5 sections from 5 mice). **d** Floating bar and circle graphs showing natural ratios of PV ( +), SST ( +), and other cell types to all GABAergic interneurons in the motor cortex of non-injected control mice. The proportions were determined by immunohistochemistry using VGAT-tdTomato mice. We examined 2,027 tdTomato ( +) cells (n = 4 sections from 4 mice). **e** Floating bar graph showing the proportions of GFP-expressing cells in PV ( +) or SST ( +) neurons. We found 733 GFP-expressing cells in 802 PV ( +) cells (91.2 ± 1.6%) and 134 GFP-expressing cells in 264 SST ( +) cells (50.7 ± 4.5%) (n = 5 sections from 5 mice). **c**—**e** Floating bars indicate upper, median, and lower quartiles (boxes) and value from each mouse (dots). Note that the majority (about 60%) of transduced cells were PV ( +) neurons (**c**). PV ( +) neurons accounted for approximately 40% of all interneurons in the motor cortex (**d**). More than 90% of PV ( +) neurons were transduced in contrast to approximately 50% of SST ( +) neurons that were not transduced (**e**)

A previous study demonstrated that mDlx enhancer transduced PV ( +) neurons and SST ( +) neurons in proportions that corresponded to their natural distributions [16]. We explored the transduced interneuron subtypes and their ratios to the total transduced cells using immunohistochemistry. Wild-type mice received intravenous infusions of AAV-PHP.B expressing GFP under the control of the mGAD65 promoter (Fig. 4a). Cerebral sections of the mice were prepared 3 weeks after viral injection, followed by immunolabeling for PV and SST. Confocal laser-scanning microscopy revealed the presence of numerous GFP-expressing neurons co-immunolabeled with PV or SST (Fig. 4b). We quantitatively assessed the ratios of PV ( +) or SST ( +) neurons to GFP-expressing neurons in the motor cortex. The results showed that PV ( +) neurons accounted for approximately 60% of the GFP-expressing neurons; SST ( +) neurons accounted for only 13% (Fig. 4c). The natural abundance of PV ( +) and SST ( +) neurons within the same region (motor cortex) of non-injected naïve VGAT-tdTomato mice, which were similarly determined by immunohistochemistry, were 40% and 20%, respectively (Fig. 4d). Thus, AAV-PHP.B carrying the mGAD65 promoter likely transduced PV ( +) neurons more preferentially than other GABAergic neuron classes, including SST ( +) neurons. Statistical analysis confirmed a significantly higher ratio of transduced PV ( +) neurons (60%) to all GFP-expressing cells than the natural distribution ratio of PV ( +) neurons (40%) to total GABAergic neurons (two-way ANOVA, with Bonferroni’s multiple comparison tests, *p* < 0.0001). To confirm the preferential transduction of PV ( +) neurons by the mGAD65 promoter, we examined the proportion of GFP-expressing cells in PV ( +) or SST ( +) neurons. The results showed that the majority (about 90%) of PV ( +) neurons expressed GFP, whereas GFP-expressing SST ( +) neurons accounted for only half (~ 50%) of the total SST ( +) neurons (Fig. 4e).

### Significant transduction of chandelier cells by the mGAD65 promoter

**Fig. 5:**
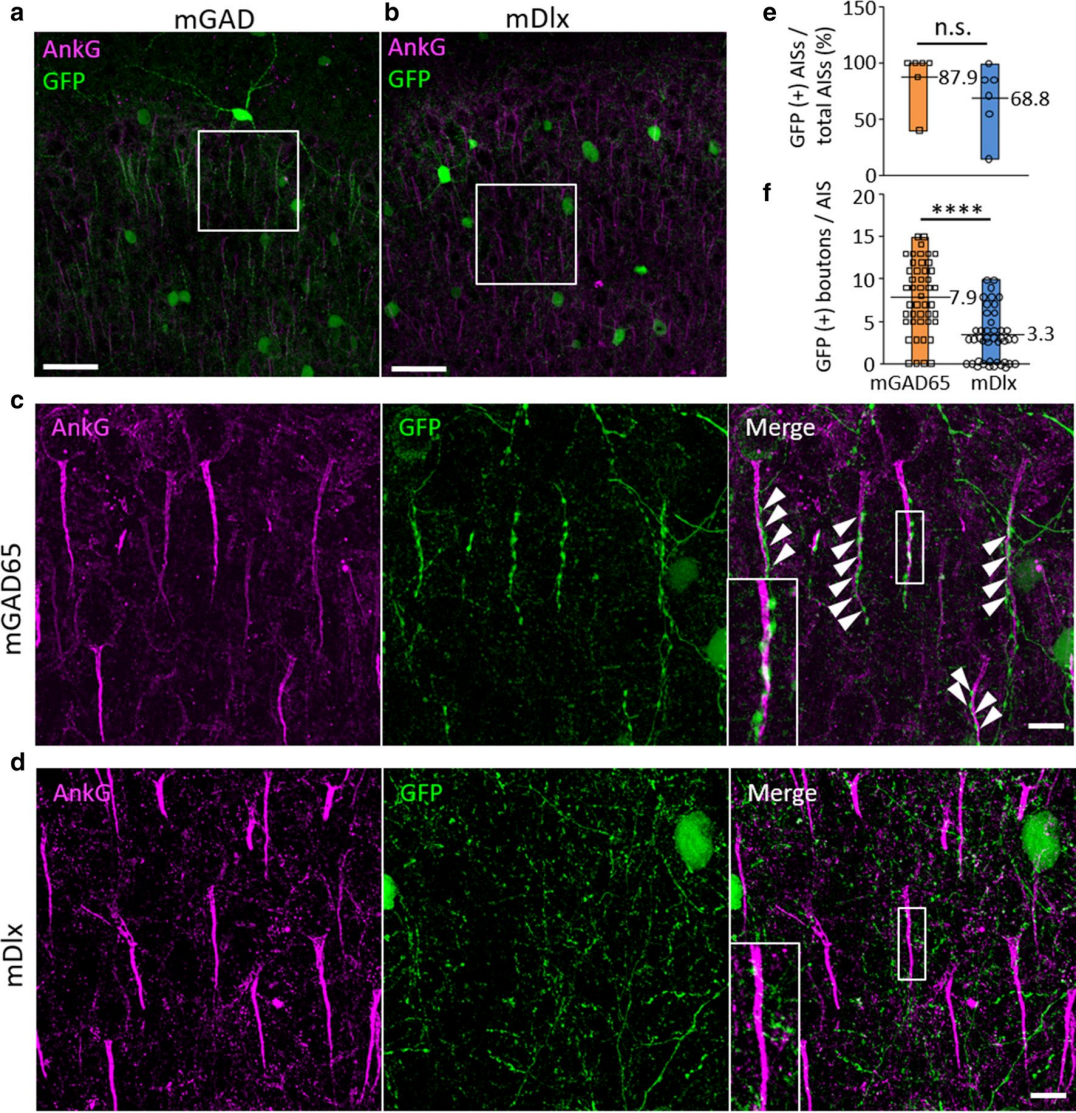
GFP labeling of the chandelier cell cartridges by the mGAD65 promoter. Immunohistochemistry of the prefrontal cortex. Sagittal sections from wild-type mice treated with AAV-PHP.B expressing GFP under the control of the mGAD65 promoter **a** or mDlx enhancer (**b**) were immunostained for ankyrin-G (magenta). **c**, **d** Enlarged images of the square regions in A, B. Note bead-like GFP labeling of chandelier cell synaptic boutons on the axon initial segments (AIS) of excitatory pyramidal neurons (arrowheads). Insets in the upper and lower right panels show the magnification of the boxes. Scale bars; 50 μm (**a**, **b**) and 20 μm (**c**, **d**). **e** The ratio of AISs with GFP ( +) boutons to total AISs in the prefrontal cortex. (F) The number of GFP ( +) boutons per AIS in the prefrontal cortex. Values adjacent to the floating boxes in (**e**, **f**) are the averages. ****p < 0.0001 on unpaired t-test

Careful observation of the transduction profile of the prefrontal cortex revealed the presence of numerous vertical dotted lines of GFP in sections transduced by the mGAD65 promoter, but not by the mDlx enhancer (Fig. 3c, d). These structures likely corresponded with axon terminals of chandelier cells (also called cartridges), which are synaptic boutons, on the AIS of pyramidal neurons [30, 31]. To verify this, brain sections expressing GFP under the control of the mGAD65 promoter or mDlx enhancer were immunostained for ankyrin G, a marker of AIS. Confocal microscopy showed numerous vertical bead-like structures of GFP along with ankyrin G immunoreactivity in the prefrontal and motor cortex transduced by the mGAD65 promoter (Fig. 5a, c), whereas the vertical GFP-labeled boutons along the ankyrin G were not obvious in the brain section transduced by the mDlx enhancer (Fig. 5b, d). These results indicate that the bead-like structures, which were clearly observed in the cortical sections transduced by the mGAD65 promoter, were chandelier-cell cartridges along the AIS.

To examine whether the mGAD65 promoter transduced chandelier cells more efficiently than that by the mDlx enhancer, we quantitatively evaluated GFP ( +) cartridges in contact with AISs. A previous study showed that the majority of chandelier cells form 3–5 axo-axonic synapses on a single AIS, and one AIS is innervated by an average of ~ 3.8 chandelier cells [30]. Thus, we examined (1) whether randomly selected AISs were labeled with GFP ( +) boutons and (2) the number of GFP ( +) boutons per AIS to estimate the number of transduced chandelier cells innervating the AIS (more transduced chandelier cells are associated with more GFP-labeled cartridges, likely resulting in more GFP ( +) boutons on the AIS).

We randomly chose 2 visual fields from layers 2 to 3 of the prefrontal cortex (total of 6 visual fields from 3 mice) and examined the presence or absence of GFP boutons along the AIS stained with ankyrin G. AISs in mice treated with AAV-PHP.B carrying the mGAD65 promoter tended to be more frequently labeled with GFP boutons (87.9 ± 9.8%) than those in mice treated with AAV-PHP.B carrying the mDlx enhancer (68.8 ± 12.5%) (Fig. 5e). Due to the large variance, however, the findings did not exhibit a statistically significant difference (*p* = 0.2564 on unpaired t-test). Next, we examined the number of GFP ( +) boutons per AIS and found significantly more GFP ( +) boutons per AIS in mice treated with AAV-PHP.B carrying the mGAD65 promoter (7.9 ± 0.6 boutons per AIS, n = 45 axons) than those in mice treated with AAV-PHP.B carrying the mDlx enhancer (3.3 ± 0.5 boutons per AIS, n = 43 axons) (*p* < 0.0001 on unpaired t-test) (Fig. 5f), suggesting that the mGAD65 promoter transduced significantly more chandelier cells than those by the mDlx enhancer.

To further confirm that the mGAD65 promoter transduced chandelier cells more robustly than the mDlx enhancer, we intravenously injected wild-type mice with a mixture of AAV-PHP.B expressing different fluorescent proteins. The mixture included AAV-PHP.B expressing GFP under the control of the mGAD65 promoter (AAV-GFP) and that expressing tdTomato under the control of the mDlx enhancer (AAV-tdTomato) with the same titer (5.0 × 10^12^ vg) (Additional file 2: Figure S2A). Three weeks after the injection of the viral mixture, brain sections were prepared and immunostained for ankyrin G. We observed numerous fluorescent protein-labeled chandelier cell cartridges under a visual field of 319.45 μm^2^. However, it should be noted that chandelier cells have multiple axon collaterals and cartridges, which are located at an average vertical distance of 360 μm from the parent soma and an average horizontal distance of 241 μm [30]. Thus, the majority of fluorescent protein-labeled cartridges present within the same visual field likely originated from the same chandelier cell. To assess cartridges from different chandelier cells, we examined only one cartridge within one randomly selected visual field (319.45 μm^2^). We first used a green channel to select one GFP-labeled cartridge, followed by a shift to a red channel to examine the co-expression of tdTomato with GFP. We observed 39 GFP-labeled cartridges in 39 visual fields from four mice, and we found that only 14 cartridges (35.9%) were co-labeled with tdTomato; the tdTomato labeling was smaller and less bright (Additional file 2: Figure S2B, C). Conversely, we used a red channel to select one tdTomato-labeled cartridge and shifted to a green channel to verify the co-expression of GFP with tdTomato. The results showed that more than 70% of tdTomato-marked cartridges were robustly co-labeled with GFP (16 cells in 22 cells from 4 mice, *p* = 0.0041 by unpaired *t*-test) (Additional file 2: Figure S2C). Certainly, we cannot rule out that the tdTomato signal was simply weaker than that of GFP under our experimental conditions. However, the brightness (product of the extinction coefficient and quantum yield) of tdTomato was 2.8 times higher than that of GFP [32], and thus, it would be reasonable to presume that the mGAD65 promoter has greater promoter activity than the mDlx promoter in chandelier cells.

### Interneuron-specific transduction in the cerebellar cortex by the GAD65 promoter

**Fig. 6:**
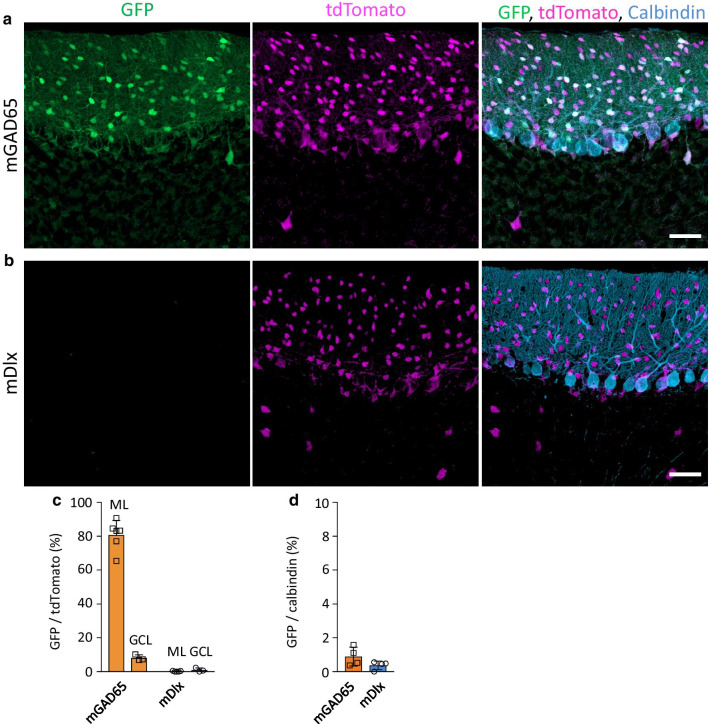
Interneuron-specific transduction in the cerebellar cortex by the mGAD65 promoter. VGAT-tdTomato mice received intravenous infusions of AAV-PHP.B expressing GFP under the control of the mGAD65 promoter or mDlx enhancer. Three weeks after the viral injection, cerebellar sections were immunostained for calbindin, a marker for Purkinje cells. **a**, **b** Representative fluorescent images of the cerebellar cortex (lobule 8) [GFP (left), tdTomato (middle), and the merged images with calbindin (blue) (right)]. Scale bars; 50 μm. **c** Graphs showing percentage of GFP ( +) cells in tdTomato ( +) cells in the molecular layer (ML) and granule cell layer (GCL). For the mGAD65 promoter, 551 tdTomato ( +) cells in the molecular layer were examined, in which 443 cells expressed GFP; in the granule cell layer, 546 tdTomato ( +) cells were observed, in which 44 cells expressed GFP (n = 6 sections from 6 mice). For the mDlx enhancer, 1061 tdTomato ( +) cells in the molecular layer were examined, in which 2 cells expressed GFP; in the granule cell layer, 495 tdTomato ( +) cells were observed, in which 4 cells expressed GFP (n = 5 sections from 5 animals). **d** Graph showing ratio of GFP ( +) Purkinje to total Purkinje cells. For the assessment of the mGAD65 promoter, we examined 1622 Purkinje cells immunolabeled for calbindin from 4 mice, in which only 15 cells expressed GFP. Similarly, for the mDlx enhancer, we observed 1451 Purkinje cells from 4 mice, and we found 6 GFP ( +) cells

We examined cerebellar slices from VGAT-tdTomato mice that received intravenous infusions of AAV-PHP.B expressing GFP under the control of the mGAD65 promoter or mDlx enhancer. The mice were sacrificed three weeks after the viral injection, and cerebellar slices were immunostained for calbindin, a marker for Purkinje cells. In contrast to the lack of GFP expression by the mDlx enhancer (Fig. 6b), highly efficient GFP expression was observed in mice treated with AAV-PHP.B carrying the mGAD65 promoter (Fig. 6a). GFP expression was almost completely matched with tdTomato expression. When examined in lobule 8, approximately 80% of tdTomato-labeled interneurons expressed GFP in the molecular layer, and approximately 10% of tdTomato-labeled interneurons expressed GFP in the granule cell layer (Fig. 6c). GFP-expressing Purkinje cells were barely detectable. Quantitative analysis showed that only 1% of Purkinje cells in the cerebellar cortex expressed GFP (Fig. 6d). GFP-positive cells in the granule cell layer were thought to be Golgi interneurons. To prove this, the slices were immunostained for metabotropic glutamate receptor 2 (mGluR2), a marker of Golgi cells, in the cerebellum [33]. Immunohistochemistry showed that all GFP-expressing cells in the granule cell layer expressed mGluR2 (Additional file 2: Figure S3).

Although Purkinje cells express GAD65 protein [34], the virally administered mGAD65 promoter failed to function in Purkinje cells. This may be due to the intravenous administration of AAV vectors; direct injection of AAV vectors into the cerebellar tissue increases the chance of viral entry into Purkinje cells, which likely causes Purkinje cell transduction. To verify this, we injected AAV-PHP.B expressing GFP under the control of the mGAD65 promoter into the parenchyma of the cerebellar cortex of wild-type mice. Three weeks after injection, the cerebellar sections were immunostained for calbindin. Confocal microscopy showed efficient GFP expression in molecular layer interneurons with a few cells in the granule cell layer (presumably Golgi cells); there was no GFP expression in Purkinje cells and granule cells (Additional file 2: Figure S4). These results confirm that the mGAD65 promoter serves as an interneuron-specific promoter in the cerebellar cortex.

### Specific transduction of GABAergic neurons in other brain regions

Using VGAT-tdTomato mice, we examined the specificity of the mGAD65 promoter in brain regions other than the cerebral and cerebellar cortices. Confocal microscopy showed colocalization of GFP with tdTomato in various brain regions such as the caudate-putamen, CA1 region of the hippocampus, anterior interposed nucleus, superior vestibular nucleus, and retina (Additional file 2: Figure S5). These results suggest that the mGAD65 promoter functions as a GABAergic neuron-specific promoter in broad CNS regions.

### Recording of Ca^2+^ activities in GABAergic interneurons of the motor cortex using the mGAD65 promoter

**Fig. 7:**
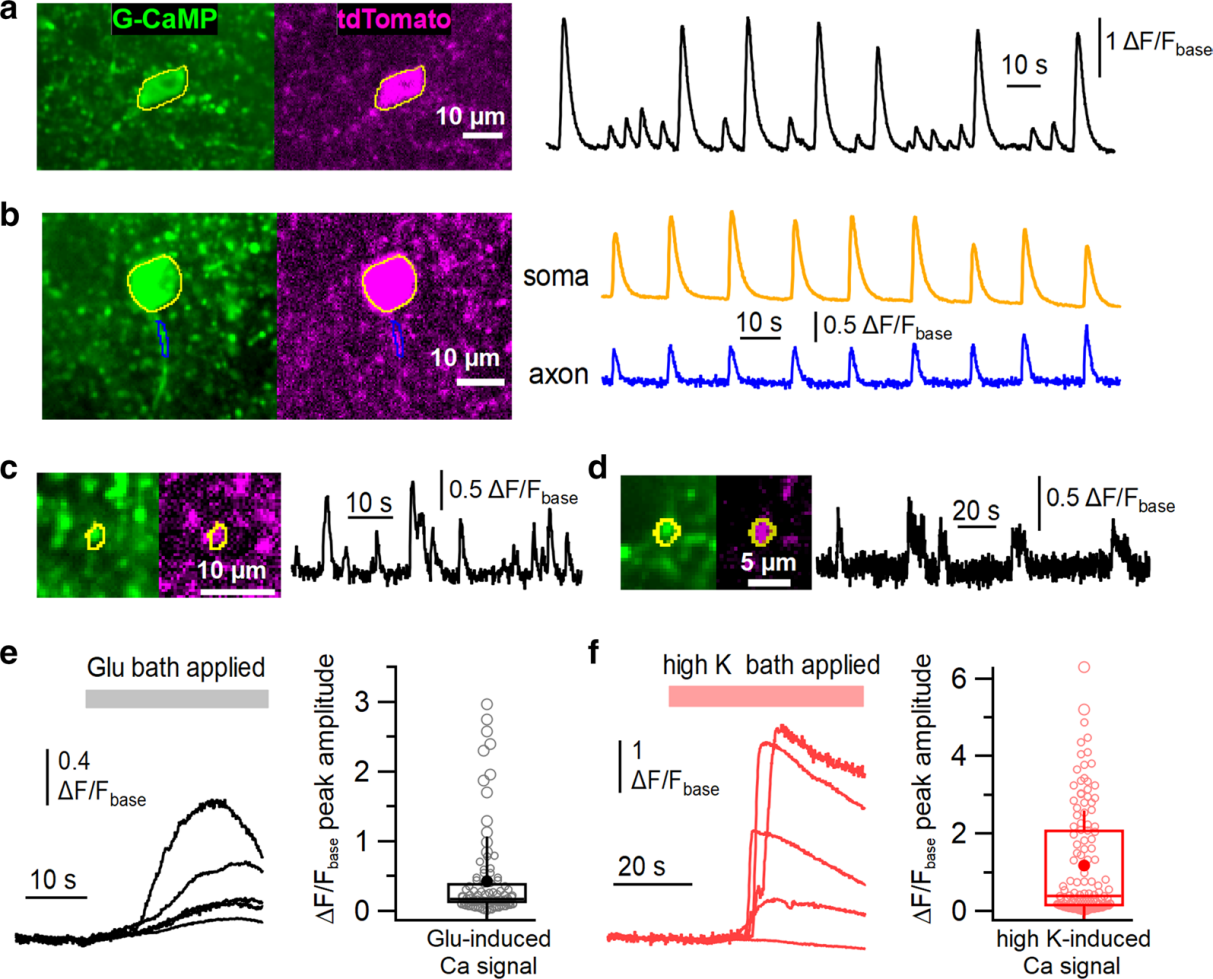
**a**–**d** Assessment of Ca^2+^ activities in GABAergic inhibitory neurons in the motor cortex using the mGAD65 promoter. Left panels show the averaged confocal images (averaging hundreds of frames) of G-CaMP signals (green) and single tdTomato images (magenta) of the same location in the M1 or M2 region of the motor cortex in the acute brain slices. Regions of interest (ROIs) were placed in somas (**a**, **b**; yellow), an axon (**b**, blue), and puncta (**c**, **d**; yellow) of the G-CaMP ( +) and tdTomato ( +) structures for Ca^2+^ signal analysis. Right panels represent traces of spontaneous Ca^2+^ signal changes calculated from the ROIs depicted in the left panels. **e**, **f** Left panels show individual traces of relative Ca^2+^ signals in several somas of GABAergic neurons in individual fields of view. Bath application of 10 µM glutamate (Glu, **e**) or high K^+^ solution (**f**) induced an increase in Ca^2+^ signals. The right panels (box plots) show open circles indicating individual data points of the Ca^2+^ signal peak values. The horizontal line and the box represent the median value and the interquartile range, respectively. The error bars indicate one standard deviation above and below the mean value (filled circle)

Next, we examined whether our GABAergic neuron-specific gene expression method using the mGAD65 promoter would function for another useful protein, which is G-CaMP7.09, a genetically encoded fluorescent calcium indicator [26]. Generally, viral expression of G-CaMP is much less efficient than GFP, and we adopted the direct AAV injection method to increase the titer of the viral solution exposed to target neurons and attain a stable and sufficient expression of G-CaMP in the motor cortex. VGAT-tdTomato mice were used in this experiment, and we confirmed that all the recorded Ca^2+^ activities in this experiment originated from GABAergic neurons by checking the colocalization of G-CaMP signals with tdTomato signals (Fig. 7a-d). Three weeks after the injection, G-CaMP was stably expressed in the motor cortex, and most of the transduced cells were tdTomato-positive. In some cells, spontaneous Ca^2+^ activity was successfully observed in cellular structures such as somas (Fig. 7a and b), axons (Fig. 7b), and puncta (putatively synaptic terminals) (Fig. 7c and d) (Additional file 3: Movie S1). The Ca^2+^ signals from small and fine structures of G-CaMP-positive cells were usually small and had compromised signal-to-noise ratios; therefore, the quantification of the Ca^2+^ signals in the subsequent analysis was performed using the data recorded from soma-like structures of G-CaMP-positive cells (Fig. 7a and b). When G-CaMP-positive neurons were stimulated using the bath application of 10 µM glutamate (which mimicked exogenous mild excitatory inputs to all the neurons in a brain slice) or a high K^+^ solution (which causes depolarization of all the neurons in the slice), Ca^2+^ signals (values of ∆F/F_base_) increased robustly (Fig. 7e and f, ∆F/F_base_ peak, Glu: 0.43 ± 0.07, n = 94 cells from 3 mice; high K: 1.18 ± 0.13, n = 126 cells from 3 mice) although the peak amplitude of the values was quite variable in the recorded cells. Collectively, these results suggest that the functional Ca^2+^ indicator G-CaMP proteins are successfully expressed in GABAergic interneurons in the motor cortex using our method. Thus, we conclude that our AAV-mediated GABAergic neuron-specific gene expression method is also applicable to the expression of a functional gene.

## Discussion

We found that a 2.5 kb genomic region upstream of exon 1 of the mouse *Gad2* gene (encoding GAD65 protein) served as an inhibitory neuron-specific promoter and that the promoter enabled us to express a functional gene in GABAergic neurons (e.g., to perform Ca^2+^ imaging in inhibitory neurons). In the cerebral cortex, the mGAD65 promoter transduced PV ( +) neurons preferentially compared to other cortical interneuron types. Notably, chandelier cells were transduced more by the mGAD65 promoter than by the mDlx enhancer.

DLX1/2 are core elements in the gene regulatory network and play critical roles in PV ( +) neuron specification [5]. However, a study using mature mice (postnatal day [P] 30) showed undetectable levels of *Dlx1* expression, in contrast to the persistent expression of *Dlx2*, in PV ( +) neurons, and only DLX2 was sufficient to drive *Gad1*, *Gad2*, and *Vgat* expression in mature mice [28]. DLX1/2 was shown to bind to the TAAT/ATTA motifs, a core sequence for homeodomain protein binding, which is located in region i and region ii in the mGAD65 promoter (Fig. 1a) [29]. Thus, in this study, the virally delivered mGAD65 promoter was likely activated in adult cortical interneurons by endogenous DLX2 binding to the motifs (region i and region ii) present in the mGAD65 promoter. Interestingly, the addition of *Gad2* exon 1 upstream of the first ATG codon to the mGAD65 promoter also conferred promoter activity on excitatory pyramidal neurons (Fig. 1). Binding of some transcription factor(s) to the additional sequence may activate the promoter in pyramidal neurons. Indeed, this ~ 500 bp region of exon 1 was shown to contain two consensus sequences of the cyclic AMP-responsive element (CRE) [35].

*Dlx5/6* genes have an inverted orientation relative to one another, and they share two intergenic enhancers in the 10 kb noncoding intergenic region 3′ to each of them [22]. We used the upstream enhancer, which is referred to as I56i [21] or mI56i, specifically bound to by both DLX1 and DLX2, as mDlx enhancers [22]. Studies using in situ* hybridization* and LacZ expression from the *Dlx6* locus showed that expression of the *Dlx6* gene was undetectable in adult cortical interneurons, while the *Dlx5*-GFP BAC transgenic mice showed robust GFP expression among different interneuron populations including PV ( +), SST ( +), neuropeptide Y( +), and calretinin( +) interneurons in the adult (P60) somatosensory cortex [36]. These results suggest that DLX5, but not DLX6, is expressed in adult mouse cortical interneurons. Thus, the mDlx enhancer, which is delivered to the mouse brain by AAV vectors, is thought to be activated by transcription factor(s) that regulate *Dlx5* expression in adult cortical interneurons. Major PV ( +) neurons are basket cells, while chandelier cells account for only a minor population [5]. Although there are no reports on the expression levels of DLX5 in mature mouse chandelier cells, weak transduction of chandelier cells by virally introduced mDlx enhancer, relative to the mGAD65 promoter (Fig. 5 and Supplementary Fig. 2), may reflect modest or only limited levels of DLX5 expression in chandelier cells.

Both GAD67 and GAD65 can synthesize the transmitter GABA. However, the two GAD isoforms have different intracellular distributions and play distinct roles. GAD65, which targets membranes and nerve endings, is likely associated with the vesicular release of GABA, while GAD67 is more widely distributed in cells, and it appears to synthesize most cytoplasmic GABA [37]. Like other cortical interneurons, chandelier cells also have GABAergic synapses with excitatory pyramidal neurons. Thus, chandelier cells synthesize GAD65 for vesicular GABA release from nerve endings. Accordingly, it is reasonable to suppose that the virally delivered mGAD65 promoter is activated in chandelier cells and drives the expression of a transgene.

Interestingly, the mGAD65 promoter was not activated in Purkinje cells, which are typical GABAergic neurons, in the cerebellar cortex, although immunohistochemistry and in situ hybridization of adult rat cerebellum revealed the expression of GAD65 protein as well as GAD65 mRNA in Purkinje cells [34]. So far, the reason for the absence of mGAD65 promoter activity in Purkinje cells remains unknown. Notably, GAD65 mRNA is not detectable until P7 in Purkinje cells [38], and neither *Dlx2* mRNA nor DLX2 protein is expressed in the adult mouse cerebellum [39]. Thus, unlike cortical interneurons, *Gad2* expression in Purkinje cells may be regulated by a postnatally upregulated transcription factor (other than DLX2), which triggers *Gad2* expression upon binding to a region outside of the mGAD65 promoter sequence.

For cerebellar cell-type-specific promoters available with viral vectors, previous studies identified the Purkinje cell-specific L7-6 promoter [13] and astrocyte-specific Gfap promoter [12, 40]. Although no cerebellar interneuron-specific promoter is currently available, the present study proposes the mGAD65 promoter as an interneuron-specific promoter when AAV vectors are directly injected into the cerebellar cortex. Thus, AAV vectors expressing a transgene under the control of the mGAD65 promoter would be useful for studying the pathophysiological roles of cerebellar interneurons and their influence on cerebellar function.

## Supplementary Information

**Additional file 1: Table S1.** Antibodies used for immunohistochemistry.

**Additional file 2: Figure S1.** Cells expressing GFP by the mGAD65 (delE1) were co-immunolabeled for GABA. A mouse received an intravenous infusion of AAV-PHP.eB expressing GABA by the mGAD65 (delE1). Three weeks after the injection, sagittal brain sections were produced and double-immunolabeled for GFP and GABA. Note that all GFP-expressing cells expressed GABA, suggesting that the mGAD65 (delE1) serves as an inhibitory neuron-specific promoter. Scale bar; 50 μm. **Figure S2.** Significant labeling of chandelier-cell cartridges by the mGAD65 promoter over the mDlx enhancer. (A) A viral mixture (equal viral titer) comprising of AAV-PHP.B expressing GFP under the control of the mGAD65 promoter and that expressing tdTomato under the control of the mDlx enhancer was intravenously injected to adult mice. (B) A representative fluorescent image of the chandelier-cell cartridge double-labeled with GFP and tdTomato. Brain sections were immunostained 3 weeks after the viral injection for ankyrin-G (blue). (C) Graph showing percentage of GFP-labeled chandelier-cell cartridges co-labeled with tdTomato and vice versa. To determine the ratio, we identified GFP-labeled cartridges along with the AIS by the GFP channel and determined whether those were co-labeled with tdTomato by the RFP channel (magenta box) (39 GFP-labeled cartridges from 4 mice were examined, in which 14 cartridges were modestly co-labeled with tdTomato) (magenta box). The green box was obtained by the opposite procedure. Namely, tdTomato-labeled cartridges were identified by the RFP channel and it was determined if they were co-labeled with GFP by the GFP channel (22 tdTomato-labeled cartridges from 4 mice were examined, in which 16 cartridges were robustly co-labeled with tdTomato). See the Materials & Methods section for more details. Floating bars indicate upper, median, and lower quartiles (boxes) and value from each mouse (dots). *p* = 0.0041 by unpaired *t*-test. Scale bar; 5 μm. **Figure S3.** Transduction of Golgi cells by the mGAD65 promoter. (A) Diagram illustrating the experimental procedure. Mice received intravenous infusions of AAV-PHP.B expressing GFP under the control of the mGAD65 promoter. Three weeks after the viral injection, the mice were sacrificed, and the cerebellar sections were immunostained for mGluR2, a marker for Golgi cells. (B) A native GFP (green) fluorescent image of the cerebellar cortex immunolabeled for mGluR2 (magenta). (C) Magnification of the box in A. Arrowheads indicate GFP-expressing Golgi cells, which were proved by mGluR2 immunolabeling. Scale bars; 50 μm (B) and 20 μm (C). IHC; immunohistochemistry. **Figure S4.** Cerebellar interneuron-specific transduction by direct parenchymal injection of AAV-PHP.B carrying the mGAD65 promoter. (A) Schematic showing the experimental procedure. (B) Immunohistochemistry of the cerebellar slice from mice 3 weeks after the viral injection. Cerebellar sections were immunostained for calbindin (magenta), a marker for Purkinje cell. Scale bar; 50 μm. IHC; immunohistochemistry. **Figure S5.** Transduction of GABAergic neurons in various brain regions by the mGAD65 promoter. Adult VGAT-tdTomato mice received intravenous infusions of AAV-PHP.B expressing GFP under the control of the mGAD65 promoter. Three weeks after the viral injection, mice were sacrificed. Confocal microscopy showed overall co-labeling of tdTomato-expressing cells with GFP in various brain regions such as the striatum (caudate-putamen), the hippocampus (CA1), the anterior interposed nucleus (IntA), the superior vestibular nucleus (SuVe), and the retina. Scale bars; 50 μm (right) and 20 μm (left).

**Additional file 3: Movie S1.** Another example of spontaneous Ca^2+^ signals observed by confocal live Ca^2+^ imaging in the motor cortex of VGAT-tdTomato mice expressing G-CaMP7.09 using our AAV-mediated GABAergic neuron-selective gene delivery method. Still image of tdTomato signals in the same field of view as G-CaMP ones is presented in a right panel in magenta. The movie plays 20 times faster than real time.

## Data Availability

All data generated or analyzed during this study are included in this published article and its supplementary information files. Please contact the authors of the materials.

## Abbreviations

GABA: Gamma-aminobutyric acid
Dlx/DLX: Distal-less homeobox
AAV: Adeno-associated virus
kb: Kilo base
GAD: Glutamic acid decarboxylase
PV: Parvalbumin
SST: Somatostatin
5HT3aR: 5-Hydroxytryptamine type 3A receptor
VIP: Vasoactive intestinal peptide
AISs: Axon initial segments
GFAP: Glial fibrillary acidic protein
CaMKII: Ca^2^^+^/calmodulin-dependent protein kinase II
BBB: Blood–brain barrier
GFP: Green fluorescent protein
VGAT: Vesicular GABA transporter
WPRE: Woodchuk hepatitis virus posttranscriptional regulatory element
HEK293T: Human embryonic kidney cells 293 cells expressing mutant version of SV40 large T antigen
SV40: Simian virus40
D-PBS: Dulbecco’s phosphate-buffered saline
PCR: Polymerase chain reaction
qPCR: Quantitative real-time polymerase chain reaction
BW: Bodyweight
Vg: Viral genome
mGluR2: Metabotropic glutamate receptor 2
ChIP: Chromatin immunoprecipitation
DNA: Deoxyribonucleic acid
bp: Base pair
ITR: Inverted terminal repeat
delE1: Deletion exon1
cDNA: Complementary deoxyribonucleic acid
CNS: Central nervous system
ANOVA: Analysis of variance
CA1: Cornu ammonis 1 of hippocampus
P: Postnatal day
CRE: Cyclic AMP-responsive element
LacZ: Bacterial beta-galactosidase gene
BAC: Bacterial artificial chromosome
SEM: Standard error of the mean
I.V.: Intravenous
RFP: Red fluorescent protein
ML: Molecular layer
GCL: Granule cell layer
IHC: Immunohistochemistry
IntA: Anterior interposed nucleus
SuVe: Superior vestibular nucleus
G-CaMP: Fluorescent calcium indicator G-CaMP7.09
M1: Primary motor cortex
M2: Secondary motor cortex
Glu: Glutamate
ROI: Region of interest
