# Supplementary material for: GABAergic neuron-specific whole-brain transduction by AAV-PHP.B incorporated with a new GAD65 promoter
Source: Mol Brain. 2021 Feb 15;14:33. doi: 10.1186/s13041-021-00746-1 (PMC7885384; doi:10.1186/s13041-021-00746-1)
Supplement: Supplementary file 1 — Additional file 1: Table S1. Antibodies used for immunohistochemistry. [file 13041_2021_746_MOESM1_ESM.pdf]

Supplementary Table

Methods		Floating (Normal)<br>Figs. 4, 6 and Supp. Figs. 2, 3, 4	Floating (CaMKII or GABA & GFP)<br>Fig. 1 and Supp. Fig. 1	Cryostat (Ankyrin G)<br>Fig. 5
**Post-fix**		4% PFA (1-overnight) at 4℃		4% PFA (4hr) at 4℃<br>25% sucrose in PBS (2-overnight)
**Brain slice**	Thickness	50 µm		20 µm
	Equipment	Vibratome (VT1000S, Leica)		Cryostat (CMS3050S, Leica)
**Permeabilization and blockade**	Blocking solution	PBS<br>5% normal donkey serum<br>0.5% tritonX-100<br>0.05% NaN_3		PBS<br>2% fish collagen peptide<br>0.2% tritonX-100
	Duration	30 min	90 min	30 min
**Primary anitibody reaction**	Antibodies (Dilution)	Goat polyclonal anti-parvalbumin (1:200)<br>Rat monoclonal anti-somatostatin (1:100)<br>Goat polyclonal anti-Ankyrin G (1:50)<br>Mouse monoclonal anti-calbindin (1:500)<br>Mouse monoclonal anti-mGluR2 (1:1000)	Mouse monoclonal anti-CaMKII (1:100)<br>Rat monoclonal anti-GFP (1:1000)<br>Rabbit polyclonal anti-GABA (1:1000)	Goat polyclonal anti-Ankyrin G (1:50)
	Solution component	PBS<br>5% normal donkey serum<br>0.5% tritonX-100<br>0.05% NaN_3	PBS<br>10% normal donkey serum<br>3% bovine serum albumin<br>0.05% NaN_3	PBS<br>2% fish collagen peptide<br>0.2% tritonX-100
	Reaction time	1-overnight at 4℃	2-overnight at room temperature	1-overnight at 4℃
**Washing before secondary antibody reaction**		PBS with 0.5% TritonX-100 (2 times)<br>PBS with 0.1% TritonX-100 (3 times)	PBS (4 times)	PBS (rinse 1 time)
**Secondary antibody reaction**	Dilution	1:1000		1:200
	Antibodies	Alexa Fluor 488-donkey anti-rat IgG<br>Alexa Fluor 568-cdonkey anti-mouse IgG<br>Alexa Fluor 594-donkey anti-rat IgG<br>Alexa Fluor 647-donkey anti-goat IgG<br>Alexa Fluor 647-donkey anti-mouse IgG	Alexa Fluor 488-donkey anti-rat IgG<br>Alexa Fluor 647-donkey anti-mouse IgG<br>Alexa Fluor 647-donkey anti-rabbit IgG	Donkey anti-Goat IgG (H+L) Highly Cross-Adsorbed Secondary Antibody, Alexa Fluor Plus 647
	Solution component	Same as the primary antibody solution		
	Reaction time	1-overnight at 4℃	6 hr at room temperature	1 hr at room temperature
**Washing before mounting**		PBS with 0.5% TritonX-100 (2 times)<br>PBS with 0.1% TritonX-100 (3 times)<br>PBS (2 times)	PBS (5 times)	
**Mounting medium**		Prolong Gold/Diamond Antifade Reagent		CC/Mountant antifade reagent

PFA: Paraformaldehyde phosphate buffer; PBS: Phosphate-buffered saline
