# Supplementary material for: GABAergic neuron-specific whole-brain transduction by AAV-PHP.B incorporated with a new GAD65 promoter
Source: Mol Brain. 2021 Feb 15;14:33. doi: 10.1186/s13041-021-00746-1 (PMC7885384; doi:10.1186/s13041-021-00746-1)
Supplement: Supplementary file 2 — Additional file 2: Figure S1. Cells expressing GFP by the mGAD65 (delE1) were co-immunolabeled for GABA. A mouse received an intravenous infusion of AAV-PHP.eB expressing GABA by the mGAD65 (delE1). Three weeks after the injection, sagittal brain sections were produced and double-immunolabeled for GFP and GABA. Note that all GFP-expressing cells expressed GABA, suggesting that the mGAD65 (delE1) serves as an inhibitory neuron-specific promoter. Scale bar; 50 μm. Figure S2. Significant labeling of chandelier-cell cartridges by the mGAD65 promoter over the mDlx enhancer. (A) A viral mixture (equal viral titer) comprising of AAV-PHP.B expressing GFP under the control of the mGAD65 promoter and that expressing tdTomato under the control of the mDlx enhancer was intravenously injected to adult mice. (B) A representative fluorescent image of the chandelier-cell cartridge double-labeled with GFP and tdTomato. Brain sections were immunostained 3 weeks after the viral injection for ankyrin-G (blue). (C) Graph showing percentage of GFP-labeled chandelier-cell cartridges co-labeled with tdTomato and vice versa. To determine the ratio, we identified GFP-labeled cartridges along with the AIS by the GFP channel and determined whether those were co-labeled with tdTomato by the RFP channel (magenta box) (39 GFP-labeled cartridges from 4 mice were examined, in which 14 cartridges were modestly co-labeled with tdTomato) (magenta box). The green box was obtained by the opposite procedure. Namely, tdTomato-labeled cartridges were identified by the RFP channel and it was determined if they were co-labeled with GFP by the GFP channel (22 tdTomato-labeled cartridges from 4 mice were examined, in which 16 cartridges were robustly co-labeled with tdTomato). See the Materials & Methods section for more details. Floating bars indicate upper, median, and lower quartiles (boxes) and value from each mouse (dots). p = 0.0041 by unpaired t-test. Scale bar; 5 μm. Figure S3. Transduction of Golgi ce [file 13041_2021_746_MOESM2_ESM.pdf]

Supplementary figure 1

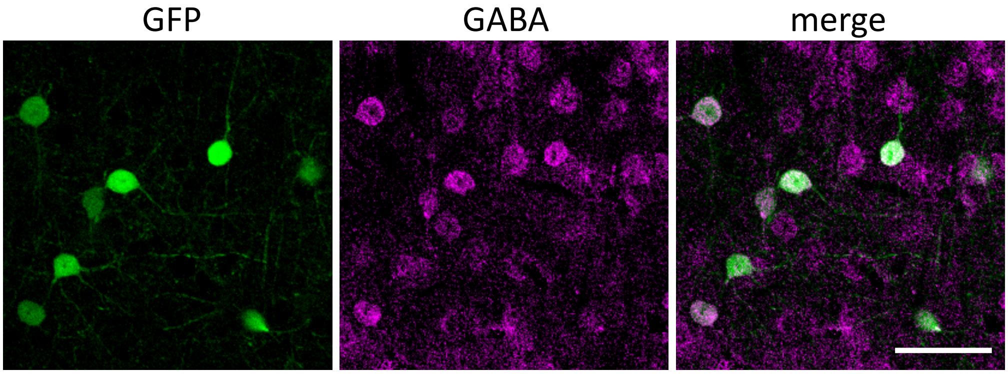

Scale bar; 50 μm

Supplementary figure 2

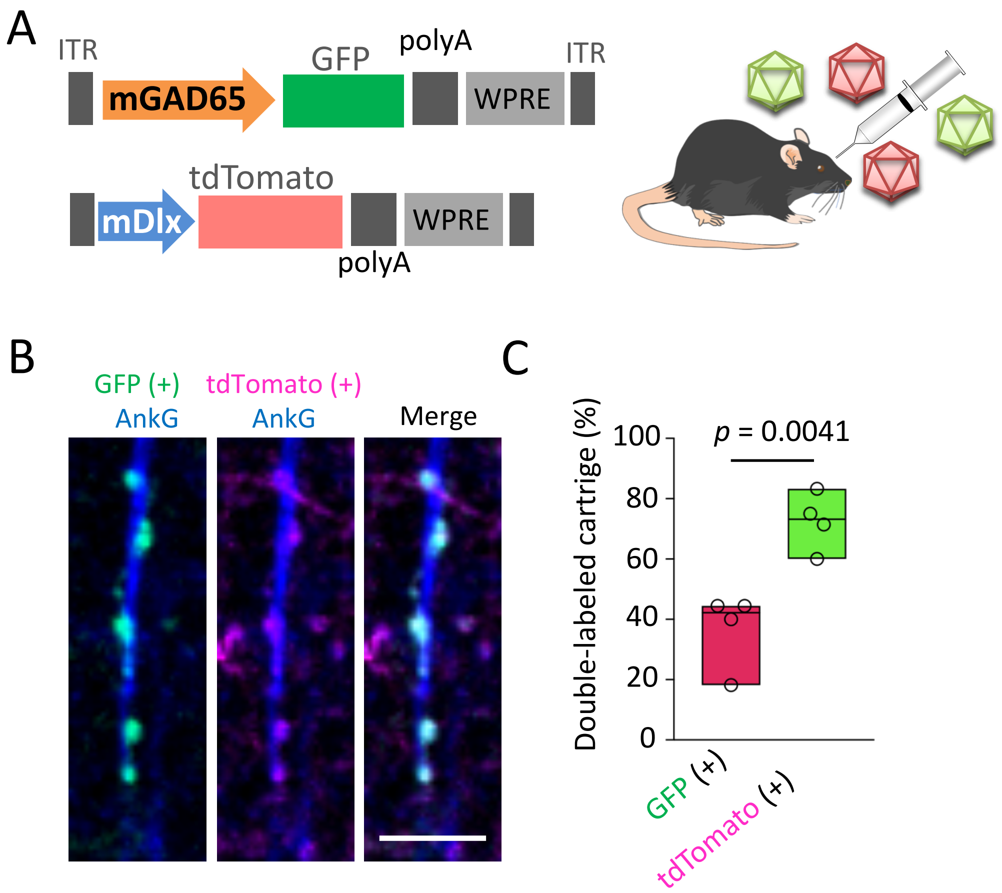

Supplementary figure 3

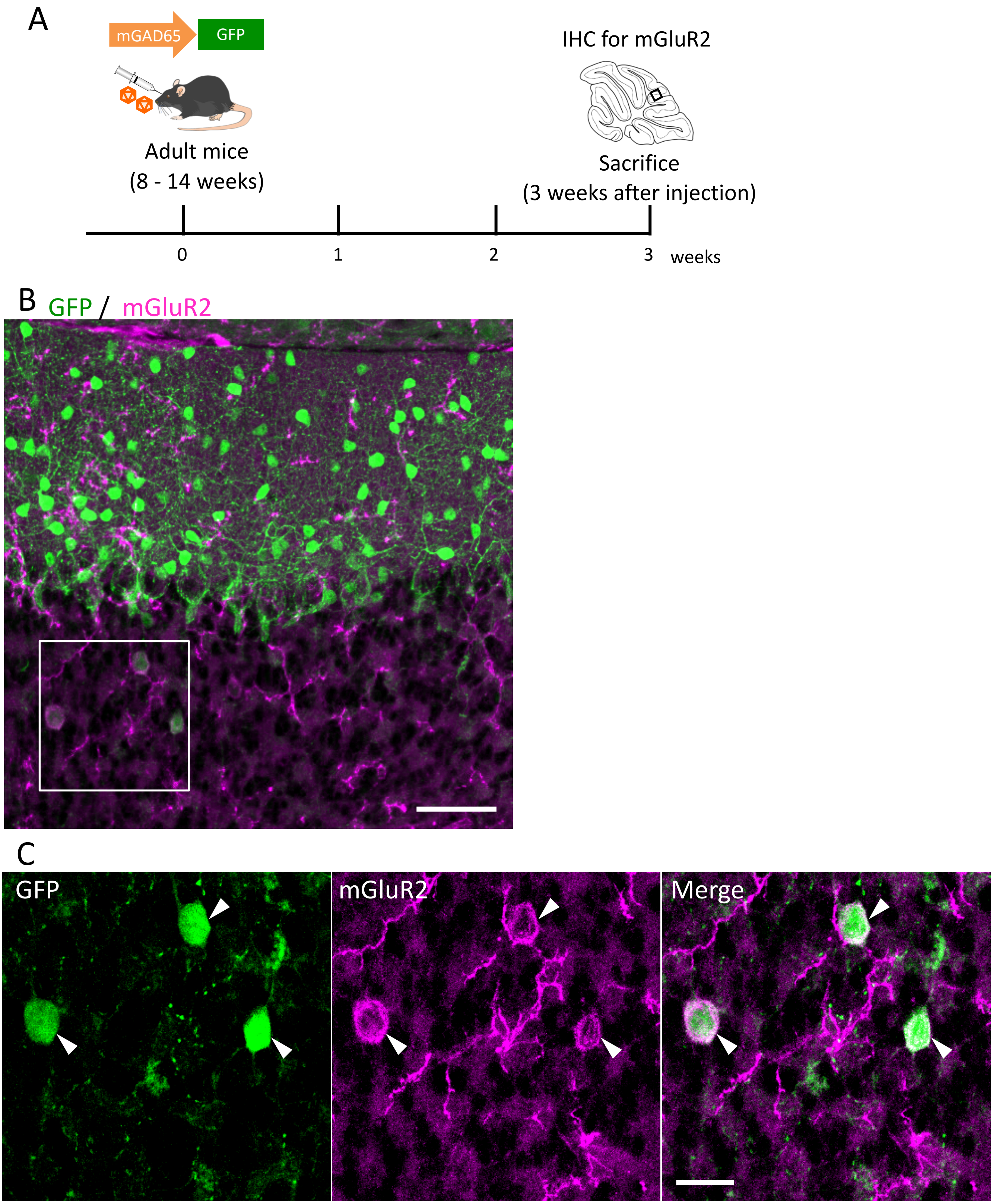

Supplementary Figure 4

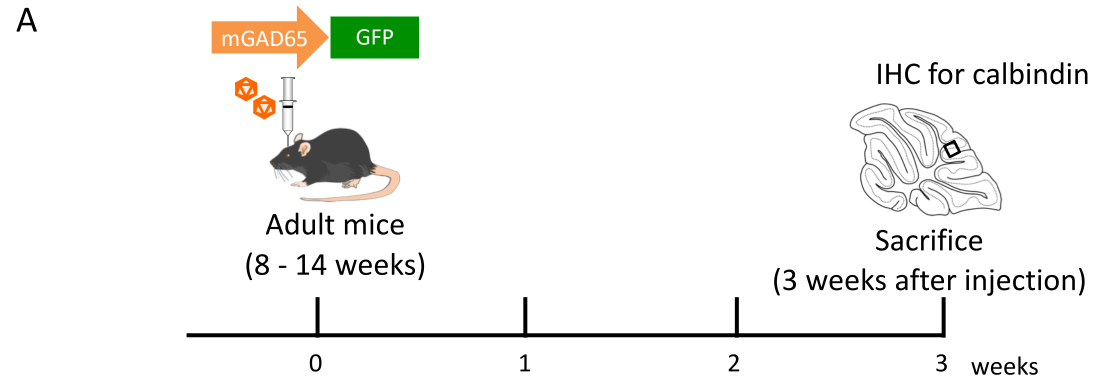

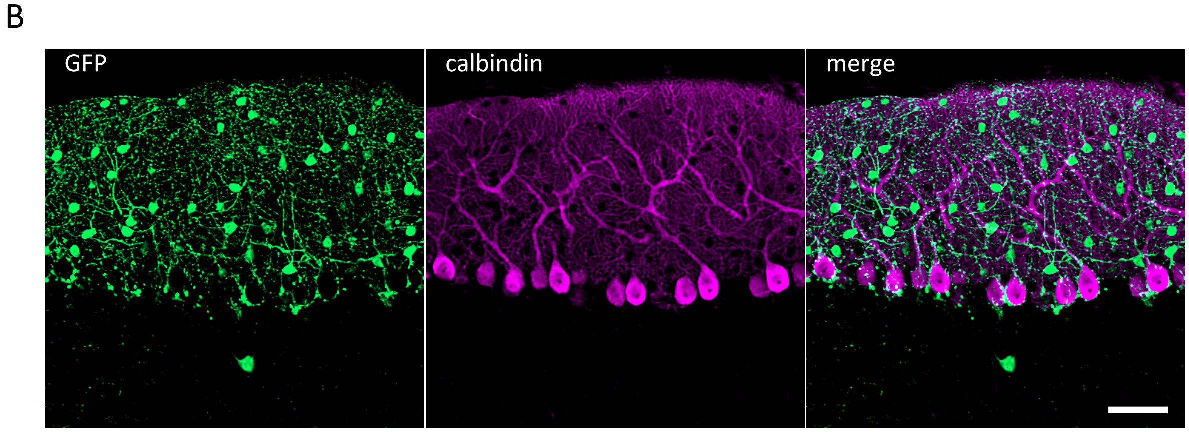

Supplementary Figure 5

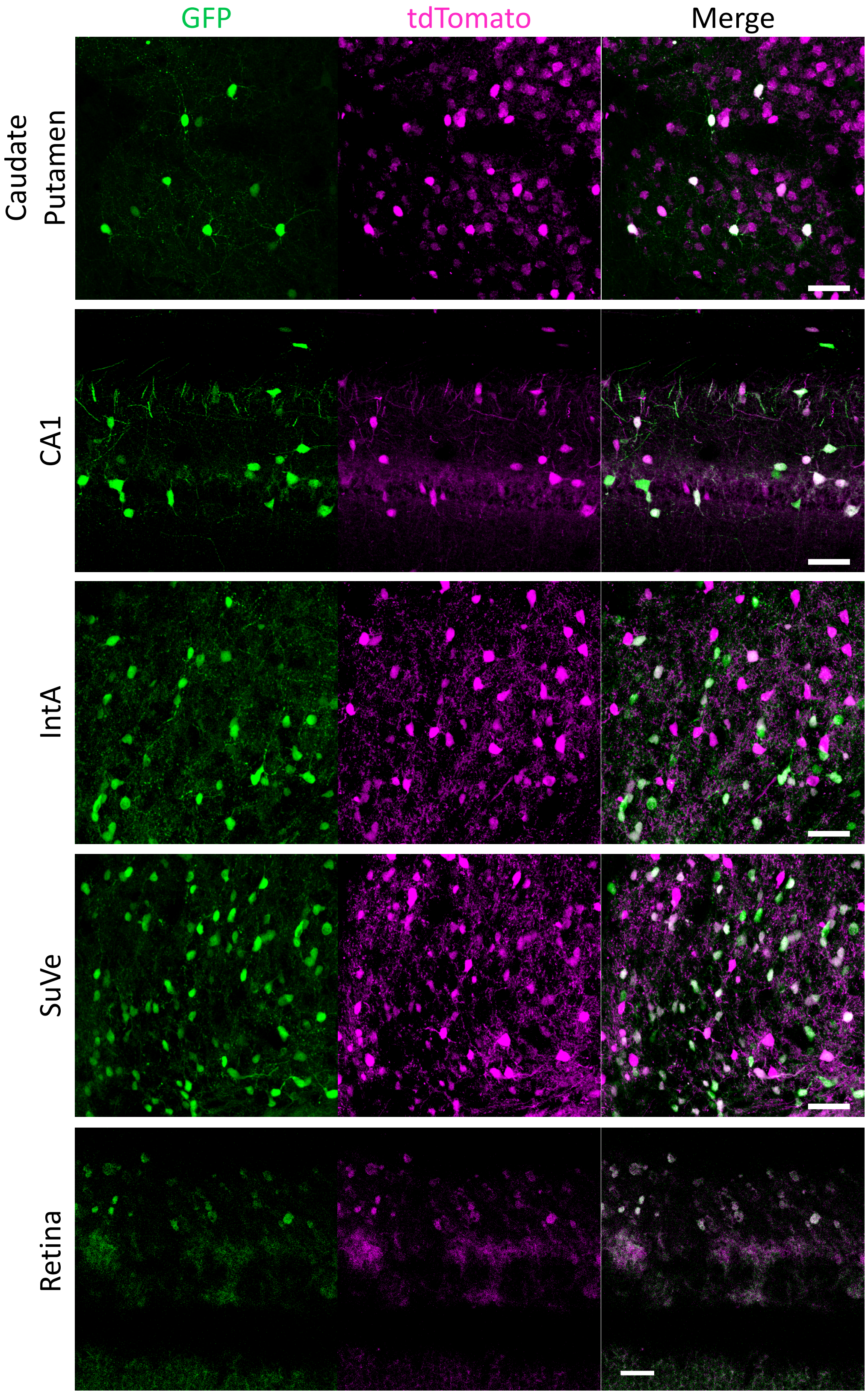

Scale bars;
50 μm(right),
20 μm(left)

Supplementary Figure Legends

Supplementary Figure 1. Cells expressing GFP by the mGAD65 (delE1) were co-immunolabeled for GABA. A mouse received an intravenous infusion of AAV-PHP.eB expressing GABA by the mGAD65 (delE1). Three weeks after the injection, sagittal brain sections were produced and double-immunolabeled for GFP and GABA. Note that all GFP-expressing cells expressed GABA, suggesting that the mGAD65 (delE1) serves as an inhibitory neuron-specific promoter. Scale bar; 50 μm.

Supplementary Figure 2. Significant labeling of chandelier-cell cartridges by the mGAD65 promoter over the mDlx enhancer. (A) A viral mixture (equal viral titer) comprising of AAV-PHP.B expressing GFP under the control of the mGAD65 promoter and that expressing tdTomato under the control of the mDlx enhancer was intravenously injected to adult mice. (B) A representative fluorescent image of the chandelier-cell cartridge double-labeled with GFP and tdTomato. Brain sections were immunostained 3 weeks after the viral injection for ankyrin-G (blue). (C) Graph showing percentage of GFP-labeled chandelier-cell cartridges co-labeled with tdTomato and vice versa. To determine the ratio, we identified GFP-labeled cartridges along with

the AIS by the GFP channel and determined whether those were co-labeled with tdTomato by the RFP channel (magenta box) (39 GFP-labeled cartridges from 4 mice were examined, in which 14 cartridges were modestly co-labeled with tdTomato) (magenta box). The green box was obtained by the opposite procedure. Namely, tdTomato-labeled cartridges were identified by the RFP channel and it was determined if they were co-labeled with GFP by the GFP channel (22 tdTomato-labeled cartridges from 4 mice were examined, in which 16 cartridges were robustly co-labeled with tdTomato). See the Materials & Methods section for more details. Floating bars indicate upper, median, and lower quartiles (boxes) and value from each mouse (dots). $p = 0.0041$ by unpaired *t*-test. Scale bar; 5 μm.

Supplementary Figure 3. Transduction of Golgi cells by the mGAD65 promoter. (A) Diagram illustrating the experimental procedure. Mice received intravenous infusions of AAV-PHP.B expressing GFP under the control of the mGAD65 promoter. Three weeks after the viral injection, the mice were sacrificed, and the cerebellar sections were immunostained for mGluR2, a marker for Golgi cells. (B) A native GFP (green) fluorescent image of the cerebellar cortex immunolabeled for mGluR2 (magenta). (C) Magnification of the box in A. Arrowheads indicate

GFP-expressing Golgi cells, which were proved by mGluR2 immunolabeling. Scale bars; 50 μm (B) and 20 μm (C). IHC; immunohistochemistry.

Supplementary Figure 4. Cerebellar interneuron-specific transduction by direct parenchymal injection of AAV-PHP.B carrying the mGAD65 promoter. (A) Schematic showing the experimental procedure. (B) Immunohistochemistry of the cerebellar slice from mice 3 weeks after the viral injection. Cerebellar sections were immunostained for calbindin (magenta), a marker for Purkinje cell. Scale bar; 50 μm. IHC; immunohistochemistry.

Supplementary Figure 5. Transduction of GABAergic neurons in various brain regions by the mGAD65 promoter. Adult VGAT-tdTomato mice received intravenous infusions of AAV-PHP.B expressing GFP under the control of the mGAD65 promoter. Three weeks after the viral injection, mice were sacrificed. Confocal microscopy showed overall co-labeling of tdTomato-expressing cells with GFP in various brain regions such as the striatum (caudate-putamen), the hippocampus (CA1), the anterior interposed nucleus (IntA), the superior vestibular nucleus (SuVe), and the retina. Scale bars; 50 μm (right) and 20 μm (left).
